# The impact of COVID-19 restrictions on eating habits, physical activity and mental health in undergraduate students of the Western Cape, South Africa

**DOI:** 10.4102/safp.v68i1.6274

**Published:** 2026-10-08

**Authors:** Shelldon A. Breda, Janicke Visser, Daniel G. Nel, Maritha Marais

**Affiliations:** 1 Department of Food Science, Faculty of Natural and Agricultural Sciences, University of the Free State, Bloemfontein, South Africa; 2 Department of Human Nutrition, Faculty of Medicine and Health Sciences, Stellenbosch University, Cape Town, South Africa; 3 Centre for Statistical Consultation, Faculty of Statistics, Stellenbosch University, Cape Town, South Africa

**Keywords:** COVID-19, student, university student, physical activity, mental wellness, mental health, eating behaviours

## Abstract

**Background:**

In response to the coronavirus disease 2019 (COVID-19) pandemic, South Africa implemented shutdowns of varying severity to mitigate the spread of the highly contagious respiratory disease. The impacts of the pandemic and other similar restriction levels were seen in tertiary schooling. The impact on undergraduate students has been noticed globally but not studied within the South African context.

**Methods:**

A self-administered electronic survey was developed by using a SUNsurvey to retrospectively investigate students’ experiences during and after the lockdown. Data were analysed to identify differences in eating behaviour, physical activity and mental health during severe restricted level (SRL) and least restricted level (LRL, *p* < 0.05).

**Results:**

Food behaviour trends were negatively impacted by the easing of restrictions as consumption of energy-dense foods increased during LRL (*p* < 0.001). Severe restricted level resulted in reduced physical activity while simultaneously increasing screen time (to > 6 h/day). The frequency of feeling stressed or anxious increased during LRL (daily: 58.6%) and had a negative impact on the mental health of students (*p* < 0.001).

**Conclusion:**

A better understanding of the impact the COVID-19 pandemic had on undergraduate students is essential. Governing bodies need to have guidelines, programmes and alternative measures in place to mitigate possible negative outcomes of similar stressful and isolating times.

**Contribution:**

Results from this study may provide insights into the eating behaviours of the said population, regardless of the pandemic status, which may prove to be insightful for universities when determining future food and physical activity intervention policies for similar future situations.

## Introduction

The World Health Organization (WHO) declared coronavirus disease 2019 (COVID-19) a global pandemic on 11 March 2020, as a result of the rapid spread and severity of infection.^1^ The South African (SA) government aimed to mitigate the spread of COVID-19 by implementing extraordinary containment measures necessitating total confinement of the population to their homes. Five levels of restriction, ranging from Level Five (Severe Restricted Level – SRL) down to Level One (Least Restricted Level – LRL), were formulated.^2^ The lockdown (SRL) commenced on 26 March 2020,^3^ which included nightly curfews, prohibition of participation in social gatherings, no access to open spaces for exercise, a total ban on trading of alcohol and closure of stores not classified as ‘essential’.^4^

The importance of maintaining a healthy lifestyle was emphasised during the COVID-19 pandemic. The WHO compiled formal publications encouraging healthy eating habits and regular exercise during this period.^5^ Still, the restrictions during lockdown impacted negatively on the eating and purchasing behaviour^6,7^ in the adult population.^6^ A marked reduction in physical activity followed the closure of recreational facilities and public spaces.^6,8^ Research shows a significant correlation between social isolation and decreased mental health^6,8^ as evidenced by an increase in overall stress and disordered eating.^6,8^ As a group of young adults, university students would be faced with similar challenges in general, but the consequences of the lockdown impacted their lives in another way.

Even without the impact of the COVID-19 pandemic, students commonly find the transition from high school to the university environment challenging.^9^ The changes in living conditions^10^ and socio-economic factors,^9,11^ as well as added academic responsibilities,^9^ cause psychological stress.^9,12,13^ During students’ first academic year, while adapting to the new environment, eating behaviours change,^9,10,14^ often leading to weight gain.^10^ Students also experience a decrease in physical activity,^9^ partly ascribed to increased sedentary habits,^12^ such as spending more time using computers.^9^ If one bears in mind the seriousness of these changes, the promotion of a healthy diet and regular physical activity among students is essential for developing lifelong habits, which contribute to a healthy body weight and overall well-being.^15^

As universities had to close their campuses because of the lockdown, students had to vacate all residences.^16^ To save the academic year,^17^ formal academic activities were replaced by the immediate implementation of online teaching wherever possible.^16^ Students faced multiple challenges and experienced a high degree of anxiety and frustration.^10^ Students were challenged by an unstable electricity supply and disrupted Internet access.^16^ Furthermore, most students seemed to lack the necessary technological skills in sharing digital content and using online collaboration platforms, as well as software and programmes required for online studies.^16^ In SA, students could return to formal lectures in 2021, when restrictions were lifted. Adjusted alert level 1 (LRL) encompassed resuming normal activities with observation of the required precautions and health guidelines.^2^

Taking measures only to prevent transmission of infectious diseases cannot be sufficient to protect individuals’ health in pandemics that can affect global health.^18^ The impact of the COVID-19 pandemic on the activity levels, mental health and education of students is well researched.^6,7,8,9,10^ Paucity of data exists about the impact of the pandemic on the eating behaviour of undergraduate students, especially in SA. To our knowledge, this is the first study conducted in the Western Cape province (SA) among undergraduate students, investigating the impact of the COVID-19 pandemic on students’ food consumption behaviours, physical activity and mental health during different levels of the lockdown (2020/2021 vs 2022).

## Research methods and design

### Study design

The study followed a descriptive, cross-sectional approach with a retrospective analytical component.

### Setting

The study focused on Stellenbosch University (SU) and the University of the Western Cape (UWC). These two universities were identified because of the differences in the nature of the campuses. Stellenbosch University is an open university, located in the town of Stellenbosch, with multiple stores, gyms and parks within walking distance. University of the Western Cape is a closed campus, with one campus gym and no official stores within walking distance.

### Study population and sampling strategy

The study population consisted of undergraduate students enrolled at either SU or UWC. All undergraduate students enrolled at these two universities were invited to participate in an electronic survey. For UWC, emails were sent internally by UWC staff. For SU, the survey was emailed electronically using the SUNSurvey distribution programme. Having participants from these two universities ensured a broad socio-economic spread. Participants who responded to advertisements distributed via the university email and provided consent by ticking the consent box constituted the study population. Only undergraduate students registered during 2020–2022 were included in the study; no postgraduate students participated.

To be included in the study, the following criteria applied:

- Undergraduate students enrolled at SU or the UWC during the years 2020–2022 (at least one of the following years: 2020/2021/2022).
- Participants who were willing to complete the survey.

Exclusion from the study applied if:

- Students who did not provide their consent.
- Students who were enrolled in postgraduate courses during the years 2020–2022 (during all 3 years mentioned).

Compared to the average postgraduate student population, undergraduate students are most likely to live alone, reside in student residences or have fewer external stressors, such as spouses, children or full-time employment. The focus of this project was limited to undergraduate students, rather than including postgraduate students, to keep the group more homogeneous.

### Sample size

The sample size was calculated by using the combined approximate number of enrolled undergraduate students in 2020/2021 (*N* = 35 000). A minimum sample size of 601 participants was calculated to be representative of the population, using a 95% confidence interval for proportion and a margin of error of 4%.

### Data collection

The researcher developed the self-administered questionnaire based on the current literature on the COVID-19 pandemic and selected guidelines for healthy eating as formulated in the Food-Based Dietary Guidelines and the WHO guidelines and definition of adequate sleep and exercise.^19^ The questionnaire content was validated by two experts in nutrition and the study supervisor. Relevant comments and/or suggestions were incorporated to improve and optimise the final product.

Created in SUNSurvey, the questionnaire comprised 78 items. Seven sections covered screening questions and consent, demographic information, eating behaviour, supplement use, physical activity, time spent on screens and mental health. After participants responded to questions about their experience during SRL, the same questions were repeated to elicit responses regarding LRL. To limit the risk of recall error, the questionnaire summarised the restrictions imposed during SRL and LRL at the beginning of the questionnaire. A pilot study conducted in the first week of October 2022 enhanced the face and content validity of the questionnaire. Furthermore, the final questionnaire underwent a round of face validity testing with five master’s students in therapeutic nutrition to assess ease of understanding and average time to completion (to prevent questionnaire fatigue).

At UWC, in accordance with its policies and distribution availability, the survey was distributed biweekly by university personnel during October 2022. At SU, it was distributed via the SUNSurvey automatic email distribution to enrolled students. At SU, the questionnaire was sent out via email by the researcher at different times, once a day, for 5 days in the last week of Oct. 2022, to increase the participation rate. A lucky draw was offered to encourage students to participate in the study and receive a R500-00 voucher.

### Data analysis

At the end of the data collection period, both university platforms provided the responses via the electronic data-capturing tool, which was automatically generated by the SUNSurvey into Microsoft Excel. The automatically provided headings were edited by the researcher to ensure that the most appropriate headings, as well as axis labels, were provided and used by the researcher and/or statistician during the production of the relevant analysis, graph and table. The statistical package used was Statistica version 14.0.

Data were automatically generated by SUNSurvey and exported into Microsoft Excel for data analysis by the research team and statistician, using the Statistical Package Statistica version 14.0. Basic descriptive statistics were utilised for the various sections of the questionnaire, such as the mean as the measure of central location for ordinal and continuous responses. Summary statistics such as standard deviations (s.d.) were utilised as indicators of spread. The strength of the relationship between continuous response variables and nominal input variables was measured with the Wilcoxon paired test. Stewart-Maxwell testing compared nominal data where three or more categories were provided. A *p*-value of *p* < 0.05 was utilised to represent statistical significance. Responses to an open-ended question about reasons causing anxiety and depression were analysed qualitatively and coded manually to identify common themes deductively.

Basic descriptive statistics were utilised, such as mean, mode and median of the various sections of the questionnaire. Summary statistics were used to describe the variables, specifically the total number of values, the minimum value, the maximum value and the s.d. corresponding to the collected data. Distributions of variables were presented with bar graphs, pie charts or tables.

Medians or means were used as the measures of central location for ordinal and continuous responses and s.d. and quartiles as indicators of spread. Relationships between two continuous variables were analysed with regression, and the strength of the relationship was measured with the Wilcoxon paired test. If a single continuous response variable was potentially related to several continuous input variables, multiple regression was used to quantify the strength of the association.

The relationship between continuous response variables and nominal input variables (such as different diets) was analysed by using the appropriate analysis of variance – the Wilcoxon paired test. Multiple comparisons were performed *post hoc* to determine where significant differences occurred among the levels of the factor(s) involved. The Wilcoxon matched-pairs test was used to compare two different variables, namely the impact during LL 5 & 4 vs LL1. A *p*-value of *p* < 0.05 was utilised to represent statistical significance throughout the analysis.

### Ethical considerations

Approval was obtained from the Health Research Ethics Committee (HREC) of the Stellenbosch University (S22/06/014-COVID-19). Institutional approval at the University of the Western Cape (UWCRP567398) was granted upon receipt of the HREC letter and the study protocol. Participation was voluntary, and anonymity was maintained as findings were presented for the entire study population.

The researcher did not have direct access to students’ email addresses prior to participants’ consent. The initial email inviting participation was sent either by the SU SUNSurvey system or by internal UWC staff. In terms of privacy, confidentiality and consent, participants’ email addresses were separated from the questionnaire prior to data analysis, for which purpose each participant received an IP (Internet Protocol) address. The email addresses were stored separately and used in the lucky draw if students provided consent to participate.

## Results

This study included 706 undergraduate students (mean age 20.9 ± 2.54 years, range 18–44 years old), who were mostly people identifying as women (63.6%, *n* = 449) and consisted of participants from SU (90.3%, *n* = 637) and UWC (9.8%, *n* = 69). The faculties with the highest percentage of participation were from Engineering (23.2%, *n* = 164), Arts and Social Sciences (19.7%, *n* = 136), Education (16.1%, *n* = 114) and Medicine and Health Sciences (15.4%, *n* = 109).

### Food consumption, eating behaviours and use of supplements

Overall, participants did not experience problems with accessibility to food (SRL: 92.9%, *n* = 656 and LRL: 93.2%, *n* = 658), which did not follow the trend of similar international studies. Approximately 37% of participants (36.5%, *n* = 258) recalled losing weight during SRL, and 40.4% (*n* = 285) gained weight when restrictions were lifted. The reported weight change during SRL and LRL was predominantly less than 5 kg (SRL: 66.3%, *n* = 460 and LRL: 68.8%, *n* = 478) (Figure 1).

**FIGURE 1:**
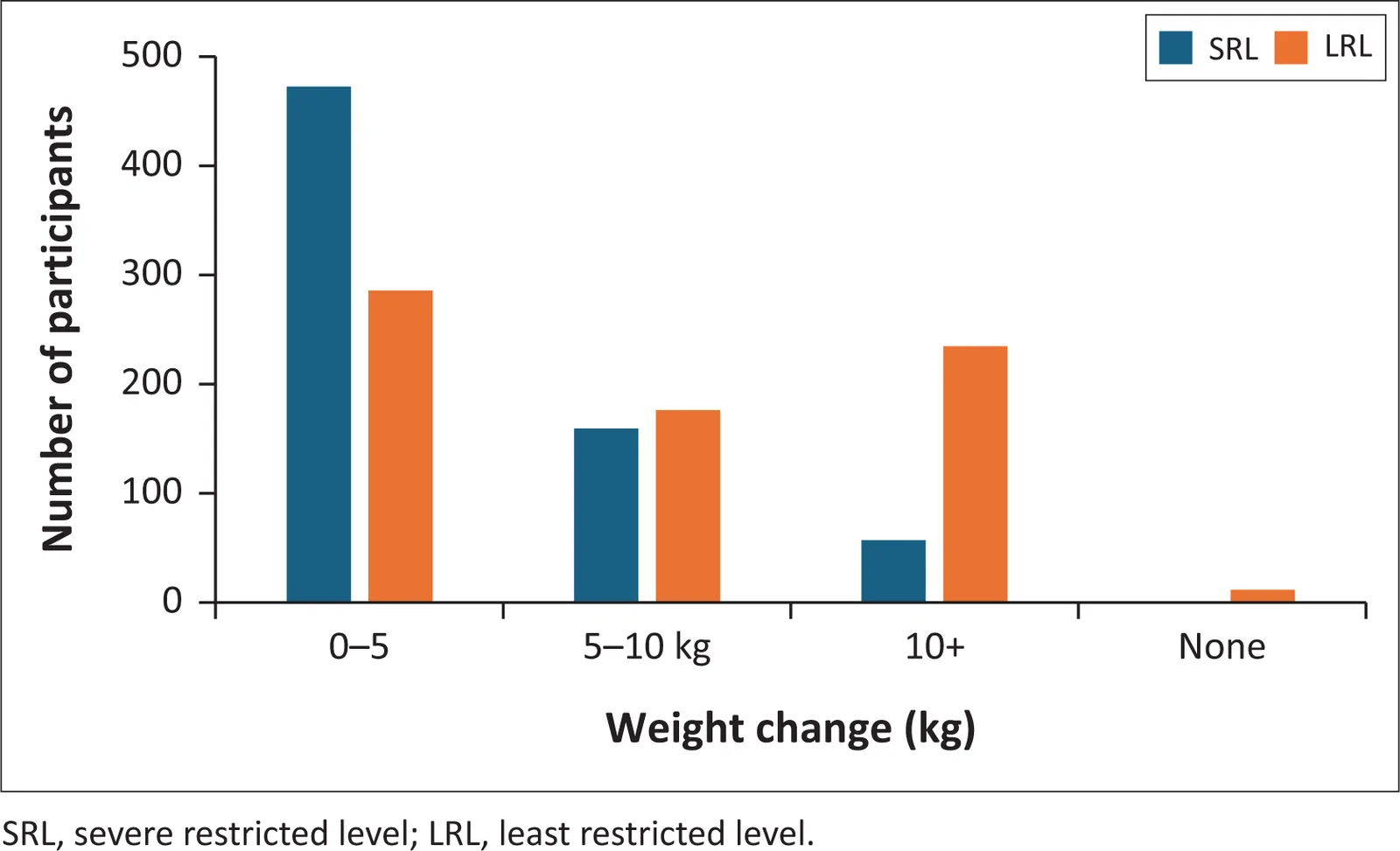
Weight change during severe restricted levels and least restricted levels of lockdown (*N* = 706).

While most participants stayed with family (90.1%, *n* = 636) during SRL, only some were responsible for purchasing (10.8%, *n* = 76) or preparing food (24.6%, *n* = 174). After SRL was lifted, 73.7% (*n* = 520) of the participants returned to living in student accommodation, where they mostly purchased (64.6%, *n* = 456) and prepared (66.3%, *n* = 468) food themselves. The SRL favoured the intake of nutrient-dense food items: fish (3–5x/week: 37.4%, *n* = 264), beans and legumes (3–5 times per week: 37.4%, *n* = 264), fruit (daily: 49.4%, *n* = 349) and vegetables (daily: 48.7%, *n* = 344), as well as dairy products (weekly: 48.6%, *n* = 343). Eating behaviour trends are presented in Table 1 by summarising the data for daily and weekly intake. For the majority of the food items, the top row for each food item reports the highest frequency of consumption during SRL and LRL, followed by daily and weekly consumption (Table 1).

**TABLE 1:** Summary of eating behaviour of undergraduate students in the Western Cape (*N* = 706).

Food item	Severe restricted level			Least restricted level			Trend (*p* < 0.05)*
	Frequency	*n*	%	Frequency	*n*	%	From SRL to LRL
Refined carbohydrates	3–5 times per week	258	36.54	6+ times per week	276	39.1	Increase
Complex carbohydrates	3–5 times per week	264	37.4	3–5 times per week	271	38.4	Same
Vegetables	1–2 times per day	177	25.1	1–2 times per day	178	25.2	Decrease
	Daily	344	48.7	Daily	303	42.9	-
	Weekly	334	47.3	Weekly	369	52.3	-
Fruit	1–2 times per day	188	26.6	3–5 times per week	183	25.9	Decrease
	Daily	349	49.4	Daily	301	42.6	-
	Weekly	293	41.5	Weekly	358	50.7	-
Beans or legumes	1–2 times per week	264	37.4	1–2 times per week	246	34.8	Decrease
	Daily	99	14.0	Daily	97	13.7	-
	Weekly	343	48.6	Weekly	316	44.8	-
Red meat	1–2 times per week	230	32.6	3–5 times per week	316	44.8	Same
	Daily	155	22.0	Daily	129	18.3	-
	Weekly	461	65.3	Weekly	463	65.5	-
Chicken	3–5 times per week	261	37.0	3–5 times per week	265	37.5	Same
	Daily	145	20.5	Daily	153	21.7	-
	Weekly	516	73.1	Weekly	508	72.0	-
Fish	1–2 times per week	349	49.4	1–2 times per week	310	43.9	Decrease
	Daily	91	12.9	Daily	97	13.7	-
	Weekly	400	56.7	Weekly	362	51.3	-
Dairy	1–2 times per day	194	27.5	1–2 times per day	179	25.3	Decrease
	Daily	367	52.0	Daily	321	45.5	-
	Weekly	292	41.4	Weekly	323	45.8	-
Water or tea or coffee	3+ times per day	232	32.9	3+ times per day	231	32.7	Same
	Daily	461	65.3	Daily	460	65.2	-
	Weekly	227	32.2	Weekly	237	33.6	-
Takeout	1–2 times per week	259	36.7	1–2 times per week	425	60.2	Increase
Sweets or chocolates	1–2 times per week	237	33.6	3–5 times per week	303	42.9	Increase
	Daily	187	26.5	Daily	173	24.5	-
	Weekly	422	59.8	Weekly	459	65.0	-
Chips	1–2 times per week	305	43.2	3–5 times per week	351	49.7	Increase
	Daily	132	18.7	Daily	111	15.7	-
	Weekly	428	60.6	Weekly	456	64.6	-
Alcohol	None	467	66.1	None	336	47.6	Increase
	Daily	31	4.4	Daily	52	7.3	-
	Weekly	188	22.6	Weekly	318	45.0	-
Sweetened drinks	None	257	36.4	None	279	39.5	Increase
	Daily	141	20.0	Daily	114	16.1	-
	Weekly	296	41.9	Weekly	313	44.3	-
Energy drinks	None	553	78.3	None	390	55.2	Increase
	Daily	64	9.1	Daily	103	14.5	-
	Weekly	80	11.3	Weekly	213	30.2	-

SRL, severe restricted level; LRL, least restricted level.

Note: ‘Daily’ includes the sum of responses to the options 1–2/day, 2–3/day and 3+/day; ‘Weekly’ includes the sum of responses to the options 1–2/week, 3–5/week and 6+/week; ‘None’ is included in neither daily nor weekly. *p values for each category were significantly below standard reporting (*p* = 0.05) at *p* = 0.00–0.0. The weekly consumption of refined carbohydrates (> 6x/week: 39.1%, *n* = 276) and energy-dense foods increased during LRL when freedom of movement was permitted. These include takeout (1–2x/week: 60.2%, *n* = 425), sweets and chocolates (weekly: 65.0%, *n* = 459), potato chips (weekly: 64.6%, *n* = 456), alcohol (weekly: 45.0%, *n* = 318), sweetened cold drinks (weekly: 44.3%, *n* = 313) and energy drinks (weekly: 30.2%, *n* = 213). The weekly consumption of complex carbohydrates (> 37%), red meat (65%) and chicken (72% – 73%), as well as daily consumption of water or tea or coffee (65%) and snacks (1–2x/day: 81%), was similar during both levels of lockdown (Table 1). The increased frequency of the consumption of takeout after the lockdown was lifted is highlighted in Figure 2.

**FIGURE 2:**
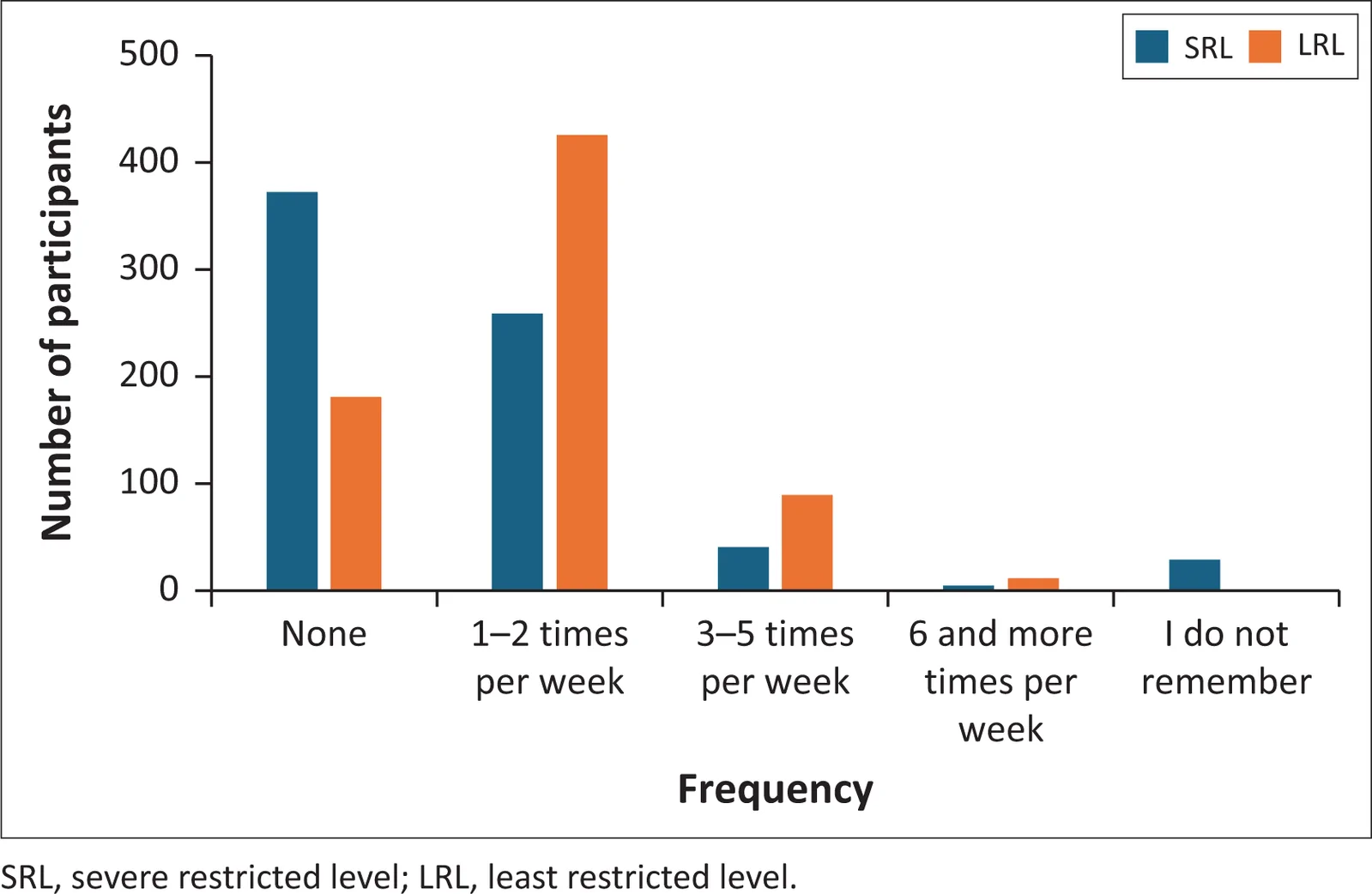
Frequency of takeout consumption during the severe restricted level and the least restricted level.

More than 50 of the participants consumed a multivitamin and/or mineral supplement (SRL: 57.4%, *n* = 405 and LRL: 55.9%, *n* = 395) or a combination of single micronutrients (SRL: 77.3%, *n* = 31.3 and LRL: 74.3%, *n* = 301). Vitamin C was the most used single micronutrient (SRL: 8.1%, *n* = 33 and LRL: 9.4%, *n* = 38). Supplements were mostly used for the general well-being of participants (SRL: 17.2%, *n* = 120 and LRL: 28.0%, *n* = 198). Family members (23.5%, *n* = 163) or the doctor (22.5%, *n* = 156) mainly provided guidance on the use of supplements during SRL. The preferred source of information during LRL was Google (31.7%, *n* = 220) or a dietitian (26.8%, *n* = 186).

### Physical activity, time spent sedentarily and quality of sleep

While participants mainly exercised 2–3 times a week or more during and after the lockdown (SRL: 37.2%, *n* = 265 and LRL: 50.1%, *n* = 354), the total number of participants doing physical activity increased significantly from 524 (74.2%) during SRL to 614 (87.0%) during LRL.

The type and duration of exercise also changed after the restrictions were lifted. During SRL, the majority of participants, who exercised, reported doing light exercises such as walking, stretching or yoga (32.7%, *n* = 231), and the majority exercised for 30–60 min (32.7%, *n* = 231). During LRL, 42.4% (*n* = 299) of participants performed a larger variety of exercises by adding jogging, cycling, running and weightlifting to light exercises, and the duration increased to > 60 min (37.5%, *n* = 265) (Figure 3).

**FIGURE 3:**
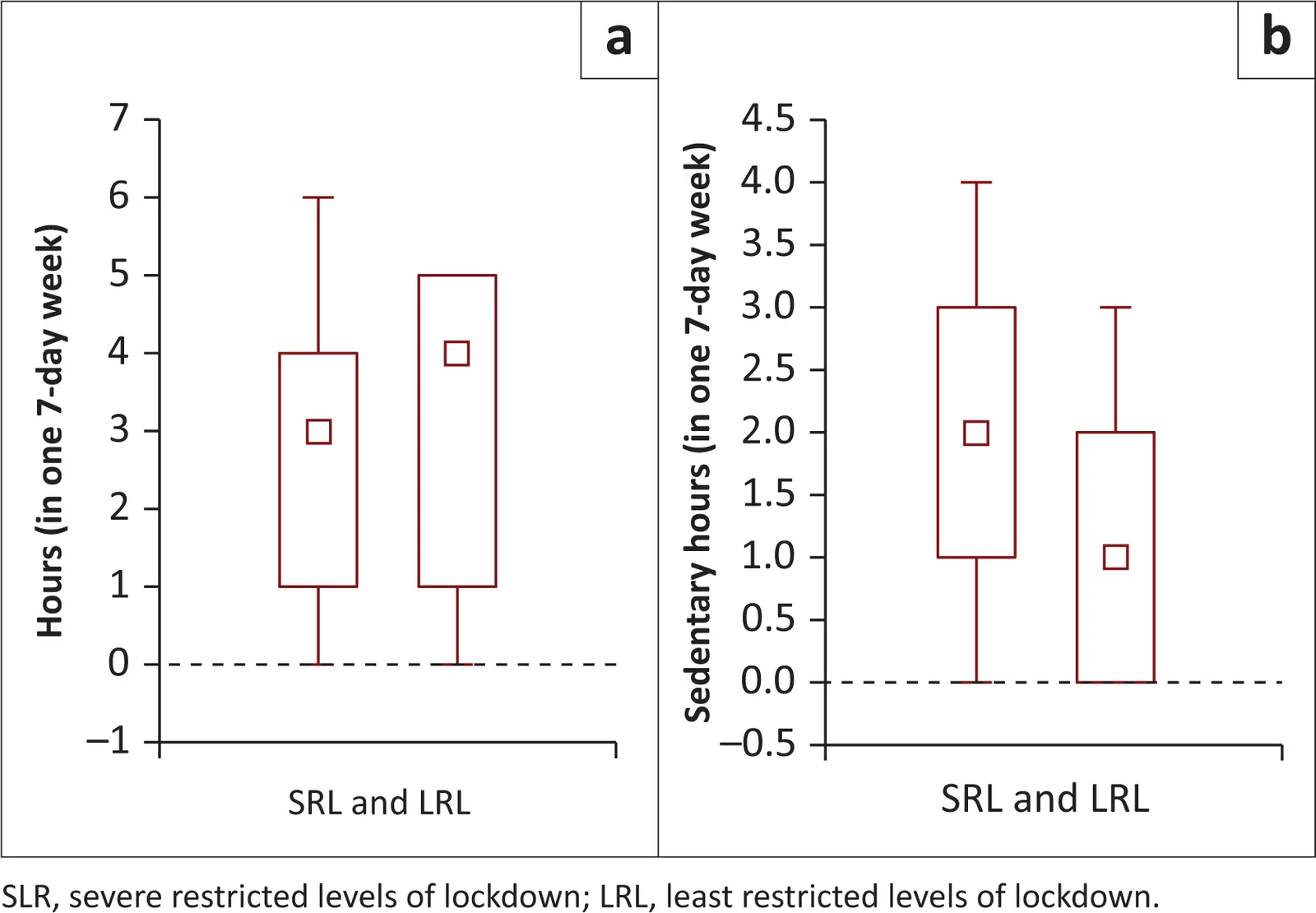
Box and whisker plot depicting the duration of exercise during SRL vs LRL (a) vs number of hours spent sedentary (b) in one 7-day week.

The level of lockdown was positively associated with the number of hours spent sedentary (> 6 h daily: SRL: 60.5%, *n* = 427 and LRL: 34.1%, *n* = 241). Wilcoxon matched-pairs test (*p* < 0.001) is also depicted in the box and whisker plot (Figure 3). Most participants spent > 6 h daily using computers, phones and tablets during SRL (76.3%, *n* = 549). The total time spent on screen significantly decreased after the lockdown (LRL: 63.6%, *n* = 449) (Wilcoxon matched-pairs test, *p* < 0.001). Furthermore, screen time was used differently after the lockdown, when more participants indicated using screen time to study > 6 h (SRL: 42.1%, *n* = 297 and LRL: 52.8%, *n* = 373). The use of social media decreased (> 6 h/day: SRL: 27.3%, *n* = 193 and LRL: 9.3%, *n* = 66), as well as time spent on screens for relaxation (SRL: > 6 h/day = 31.1%, *n* = 220 and LRL: < 3 h/day = 6.1%, *n* = 43).

The COVID-19 pandemic brought about a significant change in participants’ sleep patterns. Most participants reportedly slept for 4–6 h within a 24-h cycle during SRL (49.3%, *n* = 348) but 6–8 h during LRL (62.7%, *n* = 443) (Wilcoxon matched-pairs test, *p* < 0.001). There was a significant difference (*p* < 0.001) in the perceived quality of sleep during SRL and LRL. Perceptions ranged from ‘good – waking up feeling refreshed’ (SRL: 42.6%, *n* = 301 and LRL: 22.5%, *n* = 159) to ‘poor – restless and waking often’ (SRL: 15.7%, *n* = 111 and LRL: 23.5%, *n* = 166).

### Mental Health

Few participants (< 1%, *n* = 4) reportedly suffered from chronic mental health conditions before the COVID-19 pandemic, while negative emotions were reported by the majority of participants during and after the lockdown. Overall, feelings were indirectly related to the level of lockdown, with more participants experiencing negative emotions or the frequency thereof during LRL (Steward-Maxwell test, *p* < 0.001). A smaller number of participants felt anxious and stressed during SRL (48.2%, *n* = 340) than LRL (75.06%, *n* = 529) (Figure 4). The frequency (1 to > 3x daily) of feeling anxious, stressed, worried or depressed increased significantly after SRL (SRL: 50.5%, *n* = 356 and LRL: 58.6%, *n* = 414).

**FIGURE 4:**
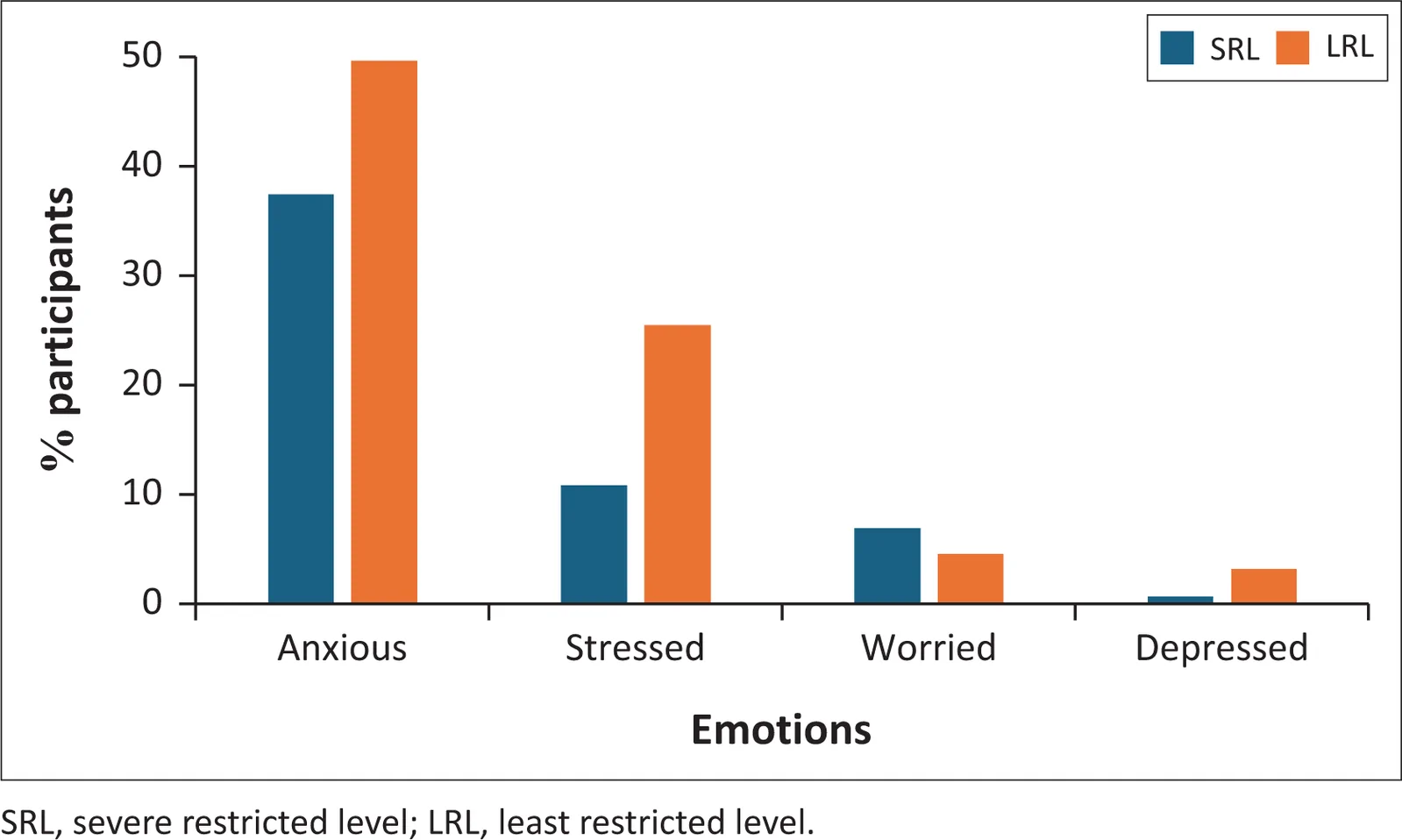
Summary of participants’ (*N* = 706) mental health during severe restricted levels and least restricted levels because of COVID-19.

Nearly two-thirds of the participants (63.3%, 447) indicated that they contracted COVID-19 during the hard lockdown. Of this group, almost 50% (*n* = 222) indicated feeling anxious (13.9%, *n* = 62), worried (7.4%, *n* = 33) or depressed (6.9%, *n* = 31).

In response to an open-ended question, participants provided valuable insight into the reasons for feeling anxious or stressed because of the lockdown. Selected quotes are provided in Table 2. They explained the impact online teaching had during the lockdown, as they suddenly had to ‘teach themselves’ even though they ‘found it difficult to understand the work’ and were ‘unable to ask questions’. They also lacked the necessary technological skills and were exposed to poor WIFI connectivity. They experienced feelings of loneliness as a result of social isolation and being without their friends, having ‘too much time alone to think’. Living at home under ‘difficult circumstances’ leads to conflict, ‘being trapped at home’, feeling judged and pressurised to perform. Students who lived on their own during the pandemic ‘had to make sure I eat and drink enough’. Some participants were eating unhealthy foods or consumed much caffeine, while others ‘had a lack of food’. Examples of direct quotes are available in Table 2. Weight loss and weight gain deepened the underlying depression they experienced. The ensuing uncertainty created feelings of being ‘out of control’, ‘unsure of future endeavours’ and a ‘fear of failing’. Having no set routine led to procrastination, unproductivity, inactivity and extended screen time (Table 2).

**TABLE 2:** Reasons for being stressed, anxious or depressed during and after the coronavirus disease 2019 pandemic.

Theme	Quote
Loneliness	‘I often felt depressed with having to sit on the couch the whole day watching TV and not being able to go out with friends’.
	‘It was isolating and exhausting. You felt cut off from people in your life, but also had very little to say to them when you did see them because not much happened. It felt like there was no point getting out of bed some days …’
	‘Being in private accommodation has played a role with my anxiety and being on my own’.
	‘I am going through a lot with no one to turn to for help, which also resulted to depression and loss of weight’.
Uncertainty	‘Being in the state of disaster opens a world filled with unknown. You lose loved ones. You’re scared that your family members would become infected. You worry about your studies, and the way to go about it is strange, different. And you have to adjust completely, which sends your mind in a spiral. You feel lost and lose sense of yourself’.
	‘During lockdown, it just felt that … that I was missing “the best years of my life,” felt a loss of purpose sitting around home, clashed with parents every day, making me feel like I was in high school again instead of independent’.
	‘It’s the amount of work that I have and constantly feeling that I am not doing good enough and that I won’t be able to finish it in time’.
	‘The weight gain caused me to feel depressed and anxious during the lockdown’.
Procrastination	‘I’m stressed-out due to assignments that I keep pushing aside. This leads to me having very unproductive periods where I take “breaks” to eat and watch things. I only procrastinate more by doing this, stressing myself out, eating and gaining weight that I really want to lose’.
	‘I felt this way because it was the first time I couldn’t distract myself from my thoughts. All I could do during that downtime was to sit and think. This brought about my depression as I had to think about things I had been avoiding by staying busy’.
	‘Was making no progress/wasting time doing nothing, just stuck in my house. Days that I was able to work on hobbies and other projects, I felt great, but on days that I just sat and watching TV or YouTube, at the end of the day, I would feel very bad’.
Online studies	‘It was a big adjustment from going to campus to basically teaching myself from home and still having to do online exams while also feeling that you don’t understand the content fully’.
	‘The online studies put a lot of pressure on me because the schedule was hectic, a lot more work than normal and a lot of adjustment’.
Inactivity	‘At the beginning of lockdown, I was very lazy and even lost the motivation to exercise, which is normally the best part of my day’.
	‘I turned to my sport as a way of relief from my depression and anxiety and even made it my identity, so when it was stripped away, I felt like I lost my sense of identity’.
	‘The depressed feeling was from not being able to move around. I tended to usually just find myself sitting in my room for entire days playing video games and watching videos. Some days not even exercising, eating, or meeting the rest of my family’.
Change	‘After lockdown, you have to change and adjust again. Change is difficult sometimes, and to constantly change, like, it takes a toll on you mentally. Everyone is moving on, but you feel stuck, and it’s so difficult to get out of bad habits. To stick to the standard the university wants you to have after a massive change in the world’.
	‘After lockdown, I had gotten so used to not doing anything, it was hard to break the habit’.
	‘The academics started off with a bang this year, and after being quite used to online education and classes, this year was a big struggle, getting back to doing well academically’.
Pressure	‘After lockdown, you have a lot more pressure to do things, and I feel like I lost my ability to handle pressure after lockdown was over’.
	‘Now I feel anxious and stressed because of the obscene amount of work some of my modules expect from me and the stress of money and how I am going to cope …’
Worried	‘I’m worried about school fees because I can’t get a bursary because of the dip in my marks that COVID-19 caused’.
	‘Work and university life and social life stress’.
	‘It is more related to stress of class and exams than anything else’.
	‘When I got here, I didn’t have funding. It was really stressful, and I couldn’t bother or worry my parents because I knew they don’t have enough money for me and the family back at home. I had to worry about meals. Sometimes, if there was nothing I could do, I would [call] them and ask for at R200 to buy food for two weeks’.

After the lockdown, participants were overwhelmed by the transition of moving back to university accommodation and attending formal lectures and found it ‘really hard to adjust’. While one participant was ‘suffocating because of too many changes’, another participant felt that ‘academics and social pressure made me lazy and demotivated’. They struggled with effective time management and the pressure of increased workload and writing exams made them feel ‘close to burnout’. The fear of failing was real. In the aftermath of the pandemic, participants expressed their concern about the lack of social distancing and not wearing masks on campus. They were worried about financial matters: ‘I can’t get a bursary because of the dip in my marks that COVID-19 caused’ (Table 2).

Because of a lack of homogeneity of variance, parametric testing was not included in this discussion.

## Discussion

The complex interchange among eating behaviours, physical activity and mental health is emphasised in the findings of this study, which focused on a group (*n* = 706) of undergraduate students in the Western Cape province of SA. During SRL, more participants exhibited a healthy food intake, but more were inclined to report sedentary habits. Contrary to findings from other studies, the consumption of energy-dense foods and feelings of anxiety, stress and depression increased after the restrictions to mitigate COVID-19 were lifted.^8,10,11,14,18,20^

### Food consumption, eating habits and the use of supplements

Of the participants in this study, 90% were living at home during SRL, where they consumed more nutrient-dense foods such as fruit and vegetables, fish, dairy products and legumes than during LRL, when they reclaimed the responsibility of their food intake. This trend follows similar international studies,^21^ wherein individuals’ food diversity reduced when attending university away from their home environment. After SRL was relaxed in SA, participants had a noticeable increase in the consumption of energy-dense foods, such as sweetened cool drinks, energy drinks, sweets, chocolates, potato chips and takeout. Time constraints^22^ and increased activity levels may encourage the consumption of takeouts.^10^ A lack of affordable healthy food options, cooking facilities or skills could also play a role similar to Yilmaz et al.’s findings.^8,10,11,14,18,20^

Current literature frequently reports weight gain among students during^18^ and after the pandemic when they had to adapt to independent living again.^9,11^ Interestingly, nearly the same number of participants in this study recalled losing weight during SRL (37%) or gaining weight after SRL (40%). The relaxation of restrictions allowed free access to outlets selling energy-dense food items and alcohol,^10,11,14^ which, combined with poor nutritional knowledge and practices,^11,14,18,22^ generally promotes an excessive energy intake.^8,14,20^ Although participants’ increase in alcohol consumption during LRL could be ascribed to increasing stress levels after SRL, the lifting of the ban on liquor trading in SA also played a major role. These trends agree with Hill et al.’s findings.

In general, students may be regarded as a vulnerable population at risk of malnutrition.^9^ As a group, they need guidance about their nutritional requirements^9^ and making informed food choices.^10^ If students had been empowered with knowledge proactively by receiving guidance about dietary habits, meal schedules, regular exercise and healthy nutrition^18^ during their first year of studies, they may have been better equipped to implement the recommendations compiled by the WHO to encourage healthy habits during isolation.^5^ Furthermore, it is suggested that interventions designed to promote healthy lifestyles in university students should consider gender, as men tend to engage in physical activity while women have greater adherence to a balanced diet.^9^

Because of the novelty of COVID-19, the public was strongly advised to increase intake of single-nutrient supplements such as zinc, selenium, vitamin D and omega-3 fatty acids without any empirical evidence of clinical benefits.^6^ It was encouraging to find that 75% of participants in this study most frequently consumed a multivitamin supplement mainly for general well-being. A recent systematic review (2021) cautioned regarding limited evidence that high-dose supplements of micronutrients will either prevent disease or promote a speedy recovery.^23^ When deciding which supplements to take, participants were more often influenced by less-informed sources (Google 32%) rather than scientific sources (dietitians 27%). During the pandemic, the Internet was inundated with often contradictory information about the importance of supplementation.^8,23^ James et al. (2021) warned that a proliferation of research related to the benefits of taking supplements does not necessarily correspond to an increase in high-quality evidence.^23^ Additionally, frequent exposure to nutrition information sources related to COVID-19 could add to the often detrimental changes in lifestyle.^6^ It is therefore crucial for dietitians to proactively and continually provide students with scientific information and nutrition education to clarify the misleading information on the Internet.^24^

### Physical activity practices

Participants reported sedentary behaviour > 6 h daily during SRL, and they preferred to use screen time for entertainment rather than for online studies. Research shows a significant (*p* < 0.05) increase in time spent sedentarily during the pandemic,^12^ and the Lancet Commission still found low participation in physical activity globally during 2022.^21^ This trend was also seen in the Naples^2^ study, as well as that of Teixeira et al. in 2021. Accordingly, the Lancet Commission commented that active recreation and transport, such as walking and cycling, should be included in future guidelines.^21^ In hindsight, it should be recommended that social distancing and exercise need to be promoted with equal vigour during periods of lockdown.^8,18^

The legally required social isolation during the lockdown^8,12,18^ led to the closure of gyms and sports facilities, thus discouraging physical activity^12,18,25^ and made it extremely difficult to reach the WHO recommendation of moderate physical activity per week for a minimum of 150 min.^26^ Participants in this study mainly performed light exercises of short duration (< 30 min/day) 2–3 times/week during SRL. Research shows that during the pandemic, exercising at home mainly included taking active breaks, doing online workouts and walking around in the home and front or backyard.^8^ A structured exercise schedule is crucial to encourage activity. With the right guidance, exercising at home could be a positive experience in which students compile a daily exercise routine to achieve their personal goals.^8^ Exercising at home has the added advantage of being performed without self-criticism or peer pressure.^8^ Social media provides a useful platform to encourage physical activity by adhering to exercise challenges and encouraging others.^8^ For example, HAN (Hogeschool van Arnhem en Nijmegen) University of Applied Sciences, encouraged physical activity during the pandemic by offering an online fitness programme using blogs and videos.^27^

### Mental health

More than half of the participants experienced negative feelings of stress and anxiety, as they were exposed to an unprecedented stressful situation of unknown duration. Psychological and physiological processes operate in tandem, and the fact that the lockdown imposed limitations on movement denied people the beneficial effect physical exercise could have had on maintaining mental health.^8,12^ Under normal circumstances, students are at risk of experiencing mental health problems and feeling lonely during the transition from school to university.^13^ Without the support of meaningful relationships, students may succumb to peer pressure, leading to several unhealthy behaviours as discussed earlier.^12^ The students participating in this study had to face a second academic transition period after the lockdown when they resumed formal lectures. Students from other countries expressed a deep concern about the challenge of timeously completing missed practicals, laboratory tutorials^28^ and internship hours after the lockdown.^29,30^ In Italy, final-year students faced a strong possibility of delayed graduation and loss of internships or job opportunities.^7^

During SRL, participants in this study slept on average 4–6 h per night, which is notably less than the 8 h deemed necessary by the National Sleep Foundation.^31^ Fear, anxiety and distress are associated with insomnia,^8^ which could have an adverse effect on mental health^25,32^ and also lead to comfort eating^8,25^ and weight gain.^32^ Student counselling programmes that include information on managing sleep problems during periods of isolation could limit stress and related consequences.^8^ By teaching students the skills of self-compassion and mindfulness, they should be able to cope better during these distressing situations.^8^

In the aftermath of the pandemic, it became clear that funding and infrastructure are required, not only for the upgrading of the necessary infrastructure to support online teaching and learning^16,17,33^ but also to provide online student mental health services.^33^ Students and lecturers need to be better equipped with the necessary skills to adapt to e-learning.^16,17,33^ The pandemic sparked an increased demand for student mental health services^33^ and a counselling approach that incorporates traditional as well as technology-based elements seems vital to cope with the demand.^18^ Low-threshold online interventions that promote help-seeking are crucial^33^ and can be individualised to accommodate students’ mental health needs within the relevant context.^34^

To summarise, Solomou et al. stated:

[*I*]nterventions to improve diet quality at the university level could reduce mental health issues; additionally, interventions to support students under stress may lead to healthier dietary habits when living on campuses.^13^

### Study limitations

As this study collected retrospective data, recall bias from the participants could exist as a result of the 2-year time lapse and variables that could not be verified. Findings from this study should be interpreted with some caution because of the uneven distribution of participants from SU and UWC and the gender imbalance. The UWC survey distribution network does not allow researchers any control over the distribution of the questionnaires. Thus, it was not possible to send reminders to encourage participation. This study provided a baseline of information, but further research will be required to establish the generalisability of the findings to other universities in South Africa.

### Recommendations

Universities in South Africa should consider the following:

- Continue to engage and utilise hybrid learning in the future, ensuring that students are accustomed to hybrid learning, should there be another pandemic that requires long-term isolation. This activity would reduce emotional and mental strain on the students by ensuring that their academic progress is not too severely impacted or halted should similar situations occur.
- Incorporate a mandatory mental and nutritional class each semester, in which experts in the topic provide techniques to prevent fatigue and provide signs or symptoms to look out for.

## Conclusion

By retrospectively collecting data, this study provides valuable information about the physiological and psychological well-being of undergraduate students during and after the COVID-19 pandemic. A participating group of undergraduate students in the Western Cape province of South Africa reported an increase in unhealthy eating behaviours, such as frequent consumption of takeout and snacking during LRL, when they had to purchase and produce meals themselves. Furthermore, limited access to gyms and recreational facilities during SRL resulted in reduced physical activity, increasing the students’ overall amount of screen time. More participants reported intense feelings of anxiety and stress upon their return to formal lectures when restrictions were lifted. Some understanding of the impact of the pandemic could prove invaluable for consideration during similar events in the future. This understanding will allow the SA government and universities to put guidelines, programmes and policies in place to mitigate possible negative outcomes while promoting the positive aspects.
